# The MANGUA Project: A Population-Based HIV Cohort in Guatemala

**DOI:** 10.1155/2015/372816

**Published:** 2015-09-06

**Authors:** Juan Ignacio García, Blanca Samayoa, Meritxell Sabidó, Luis Alberto Prieto, Mikhail Nikiforov, Rodolfo Pinzón, Luis Roberto Santa Marina de León, José Fernando Ortiz, Ernesto Ponce, Carlos Rodolfo Mejía, Eduardo Arathoon, Jordi Casabona, The Mangua Cohort Study Group

**Affiliations:** ^1^Fundació Sida i Societat, Technical Advisor Unit (UAT), Escuintla National Hospital, 5001 Escuintla, Guatemala; ^2^PhD Programme in Methodology of Biomedical Research and Public Health, Department of Paediatrics, Obstetrics, Gynaecology and Preventive Medicine, Universitat Autònoma de Barcelona, Bellaterra, 08193 Catalonia, Spain; ^3^Clínica Familiar Luis Ángel García, San Juan de Dios Hospital, Asociación de Salud Integral, 1001 Guatemala City, Guatemala; ^4^TransLab, Department of Medical Sciences, University of Girona, Girona, 17004 Catalonia, Spain; ^5^Infectious Diseases Clinic, Roosevelt Hospital, 1011 Guatemala City, Guatemala; ^6^Juan José Ortega de Coatepeque National Hospital, Coatepeque, 09020 Quetzaltenango, Guatemala; ^7^Hospital del Instituto Guatemalteco de Seguridad Social (IGSS), 1009 Guatemala City, Guatemala; ^8^National Programme for Prevention and Control of STI and HIV/AIDS (PNS), 1011 Guatemala City, Guatemala; ^9^CIBER Epidemiología y Salud Pública (CIBERESP), 28029 Madrid, Spain; ^10^Center for Epidemiological Studies on HIV/AIDS and STI of Catalonia (CEEISCAT), Institut Català d'Oncologia, Generalitat de Catalunya, Badalona, 08916 Catalonia, Spain

## Abstract

*Introduction.* The MANGUA cohort is an ongoing multicenter, observational study of people living with HIV/AIDS in Guatemala. The cohort is based on the MANGUA application which is an electronic database to capture essential data from the medical records of HIV patients in care. *Methods.* The cohort enrolls HIV-positive adults ≥16 years of age. A predefined set of sociodemographic, behavioral, clinical, and laboratory data are registered at entry to the cohort study. *Results.* As of October 1st, 2012, 21 697 patients had been included in the MANGUA cohort (median age: 33 years, 40.3% female). At enrollment 74.1% had signs of advanced HIV infection and only 56.3% had baseline CD4 cell counts. In the first 12 months after starting antiretroviral treatment 26.9% (*n* = 3938) of the patients were lost to the program. *Conclusions.* The implementation of a cohort of HIV-positive patients in care in Guatemala is feasible and has provided national HIV indicators to monitor and evaluate the HIV epidemic. The identified percentages of late presenters and high rates of LTFU will help the Ministry to target their current efforts in improving access to diagnosis and care.

## 1. Introduction

**Figure 1:**
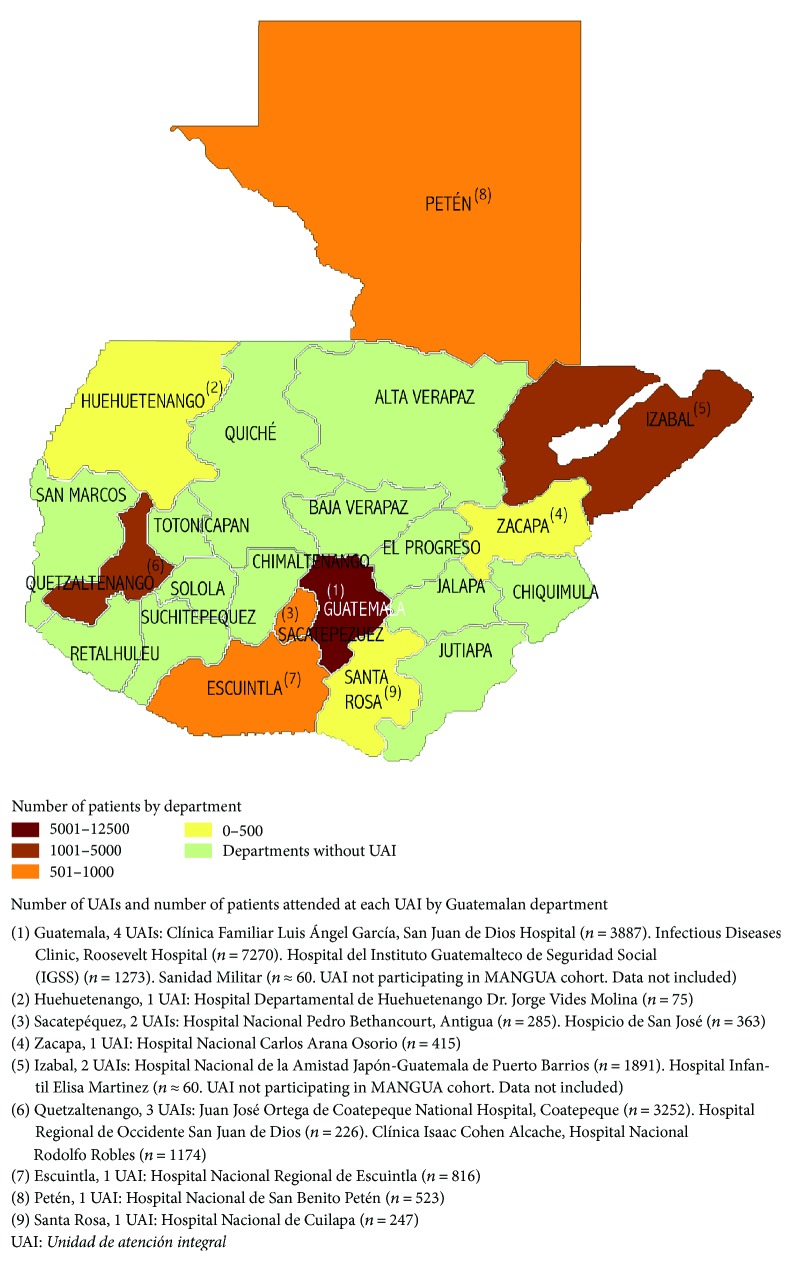
Number of UAIs and number of patients attended at each UAI by Guatemalan department.

Currently, in Guatemala the HIV epidemic is concentrated in most-at-risk populations, with prevalence between 4 and 15% among commercial sex workers and 11.5–18.3% among men who have sex with men. HIV prevalence among other vulnerable groups is 18% in people with tuberculosis (TB), 13% in prison populations, and 3.3% among youth at social risk [1–3]. During the period 2001–2005, with the support from Médecins Sans Frontières, four hospitals were providing antiretroviral treatment (ART) in Guatemala. In 2004, in alignment with the World Health Organization (WHO) 3-5 strategy and with financial aid from the Global Fund to fight AIDS, tuberculosis, and Malaria (the Global Fund), the* Ministerio de Salud Pública y Asistencia Social* (MSPAS) started to implement specialized hospital-based HIV units—*Unidades de Atención Integral* (UAIs)—across the country; therefore, extending ART coverage and increasing access to care for people living with HIV/AIDS (PLHIV) [4–6]. UAIs are administered by minimal essential staff: a physician, a nurse, a laboratory technician, and a social worker. Currently 16 UAI offer combination antiretroviral therapy (cART) free of charge (Figure 1).

In a collaborative effort between the National AIDS Program (PNS) of the MSPAS and the* Fundació Sida i Societat* (FSiS), a Catalan non-governmental organization working in global health, the HIV electronic medical record (EMR) application, MANGUA, was developed in 2006 to be implemented in all the UAIs of Guatemala. The purpose of the MANGUA application was to facilitate clinical management and provide data for national HIV indicators to the MSPAS. The MANGUA application is registered in Catalonia, Spain, and was designed and piloted with the support of the HIV department at the* Clínica Familiar* Luis Ángel García in Guatemala City. The use of the MANGUA application rapidly expanded along with the roll-out and scale-up of UAIs. MANGUA is a database application structured in query language (SQL server 2008 R2, Microsoft, Redmond, WA 98052). It was developed as an EMR to capture demographic, behavioral, clinical, and laboratory information for HIV-positive individuals enrolled in health care services in the UAIs [1, 7].

The MANGUA cohort is an ongoing multicenter, prospective observational study based on the data captured in the MANGUA application; its main objective is to generate outputs to improve both public health and clinical practice related to HIV/AIDS in Guatemala.

## 2. Methods

Currently, 14 of the 16 current UAIs participate in the cohort. 21 697 HIV-positive adults ≥16 years of age are enrolled. Data are collected by the medical staff of the UAIs following the national ethical guidelines. The cohort includes 74.3% of the cumulative number of HIV-positive patients reported to the national health authorities in 2012 (*n* = 29 211) [5]. Patients are eligible to enter the MANGUA cohort when there is confirmation of a positive HIV rapid test either with an enzyme-linked immunosorbent assay (ELISA) or a second highly specific rapid test. Discordant results are confirmed by Western blot. Patient informed consent is obtained before performing any HIV test. Participants are recruited at the UAI at HIV diagnosis, referred from primary and secondary level health care centers, or voluntary counseling and testing sites. HIV testing is repeated at the UAI for confirmation. Local policy regarding informed consent for HIV testing is applied.

Data were retrospectively collected from 2001 to 2006, and then, prospectively collected from 2006 onwards. In each UAI there is at least one data entry manager who registers routine health care data obtained from paper medical records into the MANGUA application. Retrospective clinical data dates back to the time of the patient's initial HIV diagnosis or when the clinician first visited the patient. The database is regularly updated to contain follow-up status.

Data are anonymized and sent periodically to* Unidad de Apoyo Técnico* (UAT) at the FSiS, and to the National Health Care Database (SIGSA), where data are checked for quality before returning to the UAI. All data are stripped of names and other personal identification and a unique MANGUA study code is assigned to each participant to protect confidentiality.

Every two months an interdisciplinary quality control team composed of UAT staff and data managers from PNS visits each UAI to perform source-to-database data audits. At these visits the quality control team selects at random a proportional sample of participants' medical records, such as clinical and laboratory reports, and MANGUA data are reviewed against this original documentation [8].

To guarantee the continual improvement of data validity, UAT data managers generate lists of potential errors (i.e., initiation of ART before HIV diagnosis, etc.) and check for inconsistencies and missing data [1, 9]. A MANGUA data dictionary and user manual was developed and distributed among UAI personnel. Descriptive analyses are approved by the PNS and MSPAS. A predefined set of sociodemographic, behavioral, clinical, and laboratory data are recorded at first entry to the cohort study and subsequent visits (Table 1).

Classification of AIDS-defining events is based on WHO criteria [10]. At each quarterly or semiannual follow-up visit, laboratory and clinical data are obtained and updated into the MANGUA database.

### 2.1. Statistical Analysis

Data were analyzed using STATA software version 11, (StataCorp LP, College Station, TX, USA). Sociodemographic and clinical characteristics at enrollment were compared using Pearson *χ* ^2^ test for categorical variables and Student's *t*-test for continuous variables.

## 3. Results

As of October 1st, 2012, 21 697 patients had been included in the MANGUA cohort (median age: 33 years, 40.5% female). The cohort has 45 853 person-years of follow-up, with a median follow-up time of 1.1 years. Percentages of patients with available data on the following variables are as follows: transmission route (58%), cART status at enrollment (41.1%), advanced HIV status at enrollment (50.2%), and CD4 cell counts at enrollment (56.3%). Among these patients with available data most, 92.9% (11 709/12 592), were infected by heterosexual relations. At enrollment, 89.3% (8169/9144) were naive and 74.1% (12 075/16 300) had signs of advanced HIV infection (CD4 cell counts < 350 cells/mm^3^ or WHO clinical stage 3 or 4) [10]. Only 56.3% (12 219/21 697) had baseline CD4 cell counts, of whom 63.7% (7782/12 222) had CD4 cell counts <200 cells/mm^3^ (Table 2).

During follow-up, 67.4% (14 616/21 697) started ART of which 98% started cART. At cART initiation, the median CD4 cell count was 126 cell/mm^3^ (Interquartile range [IQR]: 47–240). The treated cohort has 41 741 person-years of follow-up and a median follow-up time of 2.3 years.

Retention analysis was based on the definition described elsewhere [11, 12]. Retention proportions of living HIV-positive adults on active cART were calculated at 12 and 24 months after cART initiation. Pre- and postdecentralization periods should be differentiated. During the 2001–2005 predecentralization period the average retention was 84% and 79.7% at 12 and 24 months, respectively, with 2095 patients starting cART. For the 2006–2011 postdecentralization period, the average retention was 65.3% and 55.5% at 12 and 24 months, respectively, with 11 265 patients starting cART.

Overall, for the 11-year period 2001–2011, the average retention proportion was 73.8% and 61.5% at 12 and 24 months, respectively.

Attrition analysis was adapted from definitions described elsewhere [11, 13] and was defined as episodes of discontinuation of cART at the end of a follow-up period for the following reasons: death, loss to follow-up (LTFU), or stopping ART for any other reason while remaining in care. LTFU included the following two situations [14]. The first situation applies when patients are permanently LTFU for a 12-month period during which any additional patient visits disqualified them as permanently LTFU. In this situation, a differentiation was made between patients with no follow-up, defined as those that did not return to the clinic after the first visit scheduled for cART initiation, and patients with LTFU after starting cART, defined as those who start cART and are lost to follow-up for at least 180 days after the last visit scheduled. The second situation applies to patients LTFU who later reentered follow-up, defined as those who start cART and return for at least one visit after which the patient is not seen in the clinic for ≥180 days after the last visit scheduled but reappears at the clinic from at least day 181 onwards.

In order to calculate the global LTFU rate, the analysis took into account that a single patient might have had more than one episode of LTFU. Thus, during 41 741 person-years of follow up, 8572 episodes of LTFU (including cART stops for other reasons), were recorded with a rate of 20.5 per 100 person-years, very close to the rates seen elsewhere [14].

Attrition rates at 6- and 12-month follow-up period were studied; the 6-month interval was chosen to be consistent with the LTFU definition used; the 12-month interval was used to analyze the evolution of LTFU episodes and to be able to compare LTFU outcomes with retention rates. In the first 6 months after starting cART, 19% (*n* = 2785) of patients were lost to the program: 575 patients (20.6%) did not return to the clinic after cART initiation (no follow-up), 1568 (56.3%) were LTFU (including cART stops for other reasons), and 642 (23.1%) died. In the first 12 months after starting cART 26.9% (*n* = 3938) of patients were lost to the program: 618 patients (15.7%) did not return to the clinic after cART initiation (no follow-up), 2510 (63.7%) were LTFU (including cART stops for other reasons), and 810 (20.6%) died.

Future measurements will include an analysis of LTFU risk factors, late diagnosis, and incidence of opportunistic infections by CD4 cell counts. It is also necessary to measure death outcomes in patients starting cART and the incidence of side effects of cART.

## 4. Discussion

This is the largest cohort of HIV-positive people compiled in Central America and the cohort has a wide representation of the total HIV population registered throughout Guatemala. The MANGUA cohort has a nationwide, population-based design, with long-term follow-up. National scale-up of cART has resulted in a considerable number of patients enrolled in HIV treatment and care for HIV prevention and control in a country constrained by limited resources.

The MANGUA application was implemented soon after the scale-up of UAIs; in 2004 only 4 UAIs existed in the country; in 2008, 12 UAIs were already functioning. Scale-up of UAIs was possible because of international financial aid, mainly from the Global Fund, and the commitment of the MSPAS to decentralize HIV care in Guatemala. This scenario facilitated the acceptability of an EMR by UAI professionals, because it allowed systematization and harmonization of data collection and analysis. Comprehensive training activities were targeted to professionals, physicians, nurses, and data entry personnel, on how to use the MANGUA application. The implementation of the MANGUA cohort has been successful and has proven to be useful for both clinicians and the PNS through the provision of data for key HIV indicators to monitor the HIV epidemic in Guatemala. The MANGUA application has been integrated within the SIGSA web information system, which is crucial to ensure sustainability and strengthen HIV monitoring and surveillance in Guatemala.

Nevertheless, only approximately half of the patients registered in the cohort have data available on CD4 cell counts at baseline, partly due to under-notification and the fact that only some UAIs have available CD4 cell count analyzers. Among those with baseline data on CD4 cell counts, as much as 82.6% do not present for HIV testing until advanced symptoms of HIV infection (CD4 cell counts < 350/mm^3^). Late presenters are often more ill, have a higher mortality risk, and are less likely to respond to treatment after initiation [15–18].

**Figure 2:**
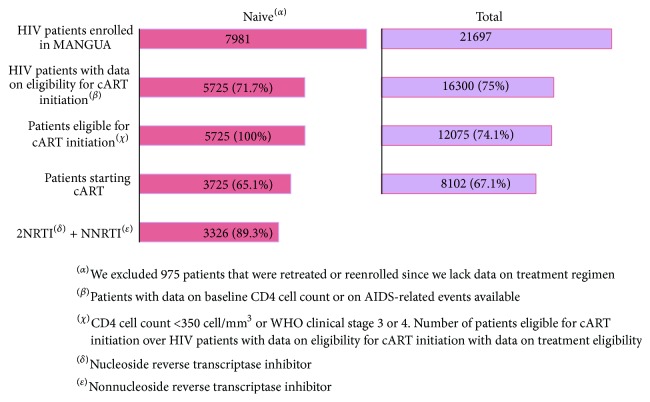
Spectrum of cART initiation in patients enrolled in MANGUA cohort.

According to the Guatemalan National Guidelines on HIV Diagnosis and Treatment, adults with WHO clinical stage disease 3 or 4 or CD4 cell counts < 350 cells/mm^3^ are eligible for cART [19]. However, 65.1% of those eligible patients do not start cART treatment and many of them are LTFU (Figure 2).

Delayed cART initiation has been reported to be the major cause of death and associated morbidity in HIV/AIDS patients [20, 21]. This fact, together with late presentation for HIV diagnosis and care and high LTFU, remains a significant challenge to efficiently prevent morbidity and mortality outcomes. It is worrisome that 30.1% (2582/8572) of patients considered LTFU return to the hospital after having interrupted ART for at least 6 months, which increases their risk of developing drug resistance or death [22–24]. Efforts should be focused on identifying reasons for LTFU with strategies to minimize it, such as creating interdisciplinary teams to investigate its underlying causes, proposing solutions that have shown to be effective in other contexts [25, 26], and expanding treatment access points in order to reduce time spent travelling to the clinic.

Retention analysis reveals that the postdecentralization period has less retention proportions than the predecentralization period. This might be explained by different factors; first, the number of HIV-positive patients starting cART in the postdecentralization period is 5.4 times higher than in the predecentralization period; and second, recently created UAIs might have less capacity to provide integrated care to HIV-positive patients, lacking the experience to effectively implement adherence and follow-up interventions.

## 5. Conclusions

Today, the MANGUA HIV cohort is a powerful tool for monitoring and evaluation as well as for research on HIV-positive patients in respect to (i) epidemiologic patterns of the HIV epidemic, (ii) delayed access to cART and burden of opportunistic infections, (iii) risk factors for LTFU and late diagnosis, and (iv) long term cART outcomes.

The strengths of this cohort include its size and the representativeness of data collected from almost all registered HIV patients in Guatemala gathered from 14 of the 16 UAIs. As a main strength, data within MANGUA covers the spectrum of engagement in care, including late HIV diagnosis, suboptimal linkage to and retention in HIV care, insufficient use of antiretroviral therapy, and suboptimal adherence to therapy [27].

In Guatemala, the AIDS surveillance system is composed of several sources of information that are not yet linked. As a result, the system is highly heterogenic and complex, making it difficult to extract data for decision making [28]. Information on quality of the AIDS surveillance system is lacking, that is, whether it is representative of the whole country and updated in a timely manner and its level of completeness and accuracy. The MANGUA database is contributing to the harmonization of HIV national surveillance data. In 2012, it gained recognition from national stakeholders and UNAIDS Guatemala as the official HIV application system. Some limitations should be noted. The MANGUA cohort has not yet reached 100% of registered HIV-positive patients declared to the MSPAS (29 211). Moreover, neither the MSPAS nor MANGUA has the ability to register HIV-positive patients from private health care centers throughout Guatemala; thus, selection bias in the MANGUA cohort might be a limitation.

The cohort is dependent on the quality of data entered by data entry managers from written medical records; therefore, long standing and skilled staff are required to maintain the quality of the clinical data, which is not always feasible. We attempt to validate clinical data and provide feedback to the UAIs in order to minimize errors and improve data recording. Nonetheless, data omissions, duplicated patient records, and incomplete and out-of-date laboratory and sociodemographic information might occur. In fact, there is a high proportion of missing data in key variables such as CD4 cell counts at baseline, treatment status at enrollment, transmission route of infection, and clinical stage category. This limits the ability to draw conclusions from the entire cohort because of data representativeness and selection bias due to differences in characteristics of patients with data available compared to patients without complete data available. In most UAIs it is not possible to maintain computerized records; clinical data are collected for administrative purposes rather than for clinical ones. Although the MSPAS is currently working through this, there is no standardized paper-based instrument shared in all UAIs to collect the essential variables recorded in MANGUA cohort, which would help in homogenizing data collection.

The MANGUA cohort is not linked with the national registry of deaths; consequently, there is little information on AIDS-related deaths, and mortality rates may be underestimated. In addition, TB and HIV services are not well integrated in Guatemala. TB screening of HIV-positive patients is poorly performed in UAIs and patients are referred to the primary health care center for TB preventive chemotherapy and treatment. Thus, data entry managers from UAIs lack information on TB treatment outcomes on HIV patients, hampering data entry into the MANGUA application from the UAIs, and underestimating TB associated incidence among HIV-positive people.

Nevertheless, the two main limitations are: first, the MANGUA application is not used directly by medical staff as an EMR in most UAIs, rather data are collected initially on paper and then digitalized. The process of informatization of the UAIs in the country may, on a medium-term basis, contribute to correct this situation. Second, high rates of LTFU may introduce selection bias due to differences in follow-up. UAI decentralization needs to be consolidated to partly overcome this problem, the stabilization of the staff in peripheral UAIs being one of its main challenges, which will assure the continuum of care and facilitate adherence to treatment.

The MANGUA cohort aims to put Guatemalan HIV/AIDS research on a par with other international collaborations in the field. This will be achieved through the development of a nationally and internationally recognized policy-relevant program of research in HIV therapy and public health and through the establishment of training and research opportunities to graduate students, postdoctoral fellowships, and clinicians across the country.

Our efforts to improve research dissemination to physicians and PLHIV as well as to improve knowledge and translational research will contribute to global recognition of the MANGUA cohort. Over the past 6 years, we have expanded our capacity to recruit participants, collect, extract, and analyze data, and engage clinicians, policy stakeholders, and funders. We are committed to share and promote MANGUA research findings to help shape programs and policy that will improve the lives of PLHIV in Guatemala and elsewhere.

## Figures and Tables

**Table 1:** Standardized variables collected in MANGUA.

Sociodemographic and behavioral variables	MANGUA study code, date of enrollment, gender, date of birth, country of origin, province of residence, ethnicity, religion, marital status, educational level, employment status, name of UAI, previous treatment, casualty date, casualty cause, cause of death, informed consent signed, vulnerability group, transmission group, probable year of infection, sexual orientation, and condom use.

Laboratory variables	Biochemistry and hematology variables, HIV rapid test, HIV ELISA test, serologies for toxoplasma, cytomegalovirus, histoplasma, cryptococcus, HBV, HCV, HSV, and syphilis. Sputum-smear microscopy for tuberculosis.

Clinical variables	WHO clinical stage, date of visit, type of visit, date of next scheduled visit, CD4 count, CD8 count, HIV RNA viral load, AIDS-related events, AIDS-defining events, and other coinfections.

Treatment variables	ART type, prophylaxis type, other treatments, initiation and stop date^*∗*^, reason for regime change^*∗*^, and adverse effects^*∗*^.

HBV: human hepatitis B virus; HCV: human hepatitis C virus; HSV: human herpes virus simplex types I and II. UAI: *Unidad de atención integral*; ELISA: enzyme-linked immunosorbent assay; RNA: ribonucleic acid; and ART: antiretroviral treatment.

^*∗*^For each type of treatment.

**Table 2:** Patient characteristics at enrollment in the MANGUA cohort.

(*n*, % of available data from variable)	Total		Men		Women		*P* value^*∗*^
	*n*	%	*n*	%	*n*	%	
Gender (*n* = 21 697, 100%)							
Age in years (*n* = 21 629, 99.7%)							<0.0001
Median, IQR	33 (27–42)						
16–24	3660	16.8	1616	44.5	2013	55.5	
25–49	15079	69.1	9182	61.4	5779	38.6	
≥50	3073	14.1	2106	68.6	964	18.6	
Ethnic group (*n* = 12 172, 56.1%)							
Ladino	9842	80.9	5881	59.8	3961	40.2	0.91
Indigenous	2330	19.1	1395	59.9	935	40.1	
Transmission route (*n* = 12 592, 58%)							<0.0001
Heterosexual	11709	92.5	6593	56.4	5102	43.6	
Homosexual	907	7.2	844	98.1	16	1.9	
Injection drug users	40	0.3	34	91.9	3	8.1	
Advanced HIV at enrollment^*∗∗*^ (*n* = 10892, 50.2%)	4168	38.3	2778	66.7	1390	33.3	<0.0001
CD4 cells/mL at enrollment (*n* = 12219, 56.3%)							<0.0001
Median, IQR	131 (46–279)						
≥350	2129	17.4	956	44.9	1173	55.1	
200–349	2311	18.9	1203	52.1	1108	47.9	
<200	7779	63.7	5208	66.9	2571	33.1	
cART status at enrollment (*n* = 8919, 41.1%)							
Naive	7981	89.5	4793	60.1	3188	39.9	0.01
Previous treatment	938	10.5	550	58.6	388	41.4	
Median (IQR) time in days between HIV diagnosis and cART initiation in days (*n* = 13195, 60.8%)	75 (27–308)	NA^*∗∗∗*^	70 (28–247)	NA	86 (26–388)	NA	0.97

^*∗*^ *P* values were calculated taking into account that transexuals (*n* = 152) were added to men for statistical convenience.

*P* < 0.05 was considered as statistically significant.

^*∗∗*^Clinical stages 3 and 4 from WHO.

^*∗∗∗*^Not applied.

IQR: interquartile range; cART: combination antiretroviral therapy.
